# A novel green synthesized ZnO-based antimicrobial nanocomposite: synergistic action, in vitro cytotoxicity, and molecular docking studies of ceftazidime, metformin, and chitosan against multidrug-resistant *Salmonella enterica*

**DOI:** 10.1186/s12934-026-02934-x

**Published:** 2026-02-14

**Authors:** Nada M. Elmayah, Mohamed I. Abou-Dobara, Zakaria A. M. Baka, Abdelaziz Elgaml, Ahmed E. Khodir, Hanaa M. Salama, Mohamed M. El-Zahed

**Affiliations:** 1 Department of Botany and Microbiology, Faculty of Science, Damietta University, New Damietta, 34517 Egypt; 2 Department of Basic Sciences, Faculty of Applied Health Sciences, Horus University-Egypt, New Damietta, 34517 Egypt; 3 Department of Microbiology and Immunology, Faculty of Pharmacy, Horus University-Egypt, New Damietta, 34518 Egypt; 4 Department of Microbiology and Immunology, Faculty of Pharmacy, Mansoura University, Mansoura, 35516 Egypt; 5 Department of Pharmacology and Biochemistry Department, Faculty of Pharmacy, Horus University-Egypt, New Damietta, 34518 Egypt; 6 Chemistry Department, Faculty of Science, Port Said University, Port Said, Egypt

**Keywords:** Multidrug-resistant *Salmonella*, Zinc oxide nanoparticles, Chitosan-based nanocomposites, Ceftazidime synergy, Molecular docking analysis, Antibacterial activity

## Abstract

**Background:**

The alarming rise of multidrug-resistant (MDR) bacteria, particularly *Salmonella* spp., has prompted an urgent search for alternative and synergistic antimicrobial strategies. In this study, a novel, green, and multicomponent nanocomposite was synthesized by integrating zinc oxide nanoparticles (ZnO NPs), chitosan (CS), the β-lactam antibiotic ceftazidime (CAZ), and the antidiabetic agent metformin (MTF) straightforward and economical manner.

**Methods and results:**

*Bacillus subtilis* strain ATCC 6633 was used to biosynthesize ZnO NPs, acting as a reliable bio-nanofactory. Various characterization techniques such as FTIR, XRD, TEM, and zeta potential analysis verified the successful integration and structural integrity of the ZnO NPs within the CS nanocomposite containing CAZ and MTF (ZnO/CS/CAZ/MTF). The FTIR spectra confirmed the presence of proteins that act as binding and supportive agents during the biosynthesis process. The produced nanomaterials have a significant positive surface charge of +28.61 mV, which enhances their stability. The particle sizes of the NPs ranged from 9.93 to 17.44 nm. The nanocomposite exhibited strong antibacterial activity against MDR *Salmonella enterica* subsp., *enterica* serovar Typhi ATCC 19214, showing a significantly increased inhibition zone of 42 mm and a greatly reduced minimum inhibitory concentration (MIC) value of 8 µg/ml, compared to the separate components. The minimum bactericidal concentration (MBC) value was found to be consistent with the MIC result, emphasizing the potent bactericidal action of the prepared nanocomposite. In silico molecular docking further supported these findings by revealing favorable interactions between the nanocomposite constituents and the outer membrane proteins (OMPs) of *Salmonella enterica* serovar Typhimurium (PDB ID: 4W4M) and *S. typhi* (PDB ID: 3UU2). Key interactions included hydrogen bonding, ionic forces, and metal coordination with critical residues. Cytotoxicity assessment using WI-38 lung fibroblast cells revealed an IC₅₀ of 84.26 µg/ml, indicating acceptable preliminary biocompatibility.

**Conclusions:**

The present study demonstrates the novelty of a ZnO-based multicomponent nanocomposite that uniquely integrates CAZ, MTF, and CS. This novel formulation exhibited synergistic antibacterial effects against multidrug-resistant *Salmonella enterica* alongside acceptable in vitro safety. The findings underscore the potential of microbially synthesized nanocomposites as promising candidates for combating antibiotic-resistant bacterial infections and support further preclinical investigations.

## Background

 The emergence of multidrug-resistant (MDR) bacteria has become a significant global health concern, threatening to the effectiveness of conventional antibiotics. The widespread misuse and overuse of antibiotics in human medicine, agriculture, and livestock production have accelerated the development of resistance mechanisms, such as genetic mutations, production of β-lactamase, and activation of efflux pump [1]. The horizontal transfer of resistance genes among bacterial species further exacerbates the problem, making infections increasingly difficult to treat [2]. MDR pathogens such as methicillin-resistant *Staphylococcus aureus* (MRSA), carbapenem-resistant Enterobacteriaceae (CRE), and *Salmonella* spp. have been linked to longer hospital stays, higher mortality rates, and significant healthcare costs [3]. *Salmonella enterica*, a Gram-negative facultative intracellular pathogen, is a leading cause of foodborne illness and systemic infections worldwide. MDR strains of *Salmonella* have emerged with resistance to first-line antibiotics such as fluoroquinolones and cephalosporins, including ceftazidime, limiting the treatment options [4]. Plasmid-encoded extended-spectrum β-lactamases (ESBLs), efflux pumps, and outer membrane modifications often mediate this resistance. These mechanisms collectively reduce the efficacy of conventional therapies.

Researchers are actively investigating innovative antimicrobial strategies to address this challenge. These strategies includes nanotechnology-based therapies, bacteriophage approaches, and drug repurposing [5–8]. Metallic and metallic oxide nanoparticles, such as Zn, Ag, Cu, Au, Fe, and Se play a crucial role in various industries, food production, medicine, agriculture, and environmental applications [9–11]. Specific bacterial-targeting NPs have been shown to decrease bacterial viability while causing minimal harm to mammalian cells. This is attributed to their enhanced ability to break down biofilms and penetrate bacterial membranes [12–14]. Zinc oxide nanoparticles (ZnO NPs) have gained considerable attention due to their inherent antimicrobial properties, primarily achieved through the generation of reactive oxygen species (ROS), disruption of bacterial membranes, and release of Zn²⁺ ion [15–17]. These mechanisms impair bacterial proteins, DNA, and cell wall integrity, ultimately resulting in cell death.

Green synthesis approaches using biological sources such as plant extracts and microorganisms have improved the safety and environmental friendliness of ZnO NPs [18, 19]. Compared to other biological processes, bacterial production is more advantageous because it allows for easier manipulation of cells and exhibits faster growth [20]. Bacteria are considered distinctive nano-factories. Bacteria can produce NPs in two ways; extracellular or intracellular synthesis. The extracellular method is advantageous because it is straightforward, inexpensive, and eliminates the need for downstream processing [21].

Two major challenges that limit the application of nanometals in bacterial treatment are poor stability and particle aggregation. Potential strategies to overcome these issues include incorporation into polymers and the formation of composites [22, 23]. Chitosan (CS), a cationic biopolymer derived from chitin, enhances antibacterial effects by interacting with negatively charged bacterial membranes, increasing permeability, and promoting cell lysis [24, 25]. Additionally, CS has shown potential as a drug carrier, improving the bioavailability of antibiotics and nanoparticle stability [26]. When combined with ZnO nanoparticles and conventional antibiotics, it exhibits enhanced synergistic effects, offering a promising strategy against multidrug-resistant (MDR) bacterial strains [27, 28].

Ceftazidime (CAZ), a third-generation cephalosporin, is widely used to treat Gram-negative bacterial infections. It works by inhibiting bacterial cell wall synthesis through binding penicillin-binding proteins (PBPs). However, its efficacy has been limited by increasing resistance due to ESBLs and β-lactamase enzymes, especially in *Enterobacteriaceae* and *Salmonella* spp [29, 30]. On the other hand, metformin (MTF), traditionally used as a first-line treatment for type 2 diabetes, has recently been investigated for its non-antibiotic antibacterial potential. It has shown the ability to alter bacterial membrane permeability, disrupt glucose metabolism, and enhance host immune responses, thereby enhancing the activity of conventional antimicrobials [31, 32]. Due to the limitations of monotherapy, there is a growing interest in utilizing multicomponent antimicrobial systems, which incorporate NPs, biopolymers, and repurposed drugs. However, the integration of bio-synthesized NPs with non-antibiotic metabolic modifiers like MTF remains largely unexplored.

The strength of this study lies in its multi-targeted approach to overcoming MDR *Salmonella* resistance and investigating the synergistic antibacterial activity of a novel, environmentally friendly ZnO-based nanocomposite incorporating CS, CAZ, and MTF. This quadruple-action formulation is designed to penetrate bacterial membranes, inhibit cell wall synthesis, and disrupt metabolic homeostasis simultaneously. The antibacterial action of the ZnO/CS/CAZ/MTF nanocomposite against multidrug-resistant *Salmonella enterica* was investigated using agar well diffusion, minimum inhibitory concentration (MIC), and minimum bactericidal concentration (MBC) assays. This work also offers unique structural insights into the computational docking and molecular interactions of ZnO NPs, CS, and CAZ with the outer membrane proteins (OMPs) of *S. enterica* serovar Typhimurium (PDB ID: 4W4M) and *S. typhi* (PDB ID: 3UU2). By combining multiple antibacterial mechanisms into a single formulation, this research presents a promising and innovative strategy for high-priority infections where conventional therapies have failed.

## Materials and methods

### Materials

Chitosan (w/v, MW < 100 kDa, deacetylation degree: Min. 90%) was purchased from Oxford Lab Fine Chem LLP, India. Zinc nitrate hexahydrate salt was purchased from Techno Pharm-chem, India. MET was commercially purchased from Sigma Chemical Company, St. Louis, MO, USA. The human lung fibroblast cell line (WI-38) was obtained from ATCC through the Holding company for biological products and vaccines (VACSERA) in Cairo, Egypt. RPMI-1640 medium, MTT, and DMSO were purchased from Sigma Co. in St. Louis, USA, while Fetal Bovine serum was obtained from GIBCO in the UK. The nutritional media used were acquired from Oxoid Ltd. in England. The standard strain *Bacillus subtilis* ATCC 6633 and the MDR *Salmonella* subsp. *enterica* serovar Typhi ATCC 19214 were provided by the Microbiology Laboratory at the Faculty of Science, Damietta University in Damietta, Egypt.

### Extracellular biosynthesis of zinc oxide nanoparticles

A modified approach based on Hamk et al. [33] was used for the extracellular synthesis of ZnO NPs using the cell-free supernatant of *Bacillus subtilis* ATCC 6633. The bacterial strain was cultured in nutrient broth and incubated at 37 °C, 150 rpm for 24 h. Following centrifugation (5000 rpm, 20 min) and filtration (0.2 μm), the supernatant was mixed with a 6 mM zinc nitrate solution (1:1 v/v%). The pH of the reaction was adjusted to 9–10 using 1 mM NaOH, and the mixture was left to incubate overnight under the same conditions. A color change to yellowish white indicated the formation of ZnO NPs. The NPs were collected by centrifugation, washed with distilled water, oven-dried at 80 °C for 8 h. and calcined at 550 °C for 3 h. before further work and characterization.

### Preparation of ZnO NPs/ceftazidime/metformin/chitosan nanocomposite

Chitosan (2 mg/ml) was dissolved in 0.5% (v/v) acetic acid while stirring until a homogeneous solution was formed. Biosynthesized ZnO NPs (100 mg/ml) were then gradually added with continuous stirring to ensure uniform dispersion. The pH was adjusted to 5.5–6.0 to enhance the interaction between ZnO NPs and CS. Next, MTF (0.01 mg/ml) was introduced and allowed to interact, followed by the addition of CAZ (1 mg/ml) under continuous stirring to achieve a well-dispersed complex. The mixture was stirred for several hours at room temperature and left overnight without agitation to ensure stability [34]. The resulting nanocomposite was purified via centrifugation (10,000 rpm, 15 min, 4 °C), washed with double-distilled water, and freeze-dried at − 50 °C for storage.

### Characterization of the biosynthesized nanomaterials

A comprehensive characterization was conducted to obtain a detailed understanding of the structural, morphological, and physicochemical properties of the synthesized nanomaterials. UV-Visible spectroscopy (UV-VIS, V-630, Japan) was utilized to verify the formation and optical properties of ZnO nanoparticles and their nanocomposites. Fourier transform infrared spectroscopy (FTIR, FT/IR-4100 type A) was used to identify functional groups and evaluate molecular interactions between ZnO NPs, CAZ, MTF, and CS. X-ray diffraction (XRD, LabX XRD-6000, Shimadzu, Japan) was employed to determine the crystalline structure and phase purity of the composite. Transmission electron microscopy (TEM, JEOL JEM-2100, Japan) provided detailed information on particle size, shape, and dispersion of ZnO NPs within the composite. Zeta potential analysis (Malvern zs90, UK) was conducted to evaluate surface charge and colloidal stability. Together, these techniques offered a comprehensive insight into the physicochemical characteristics of the prepared nanocomposite.

### Antibiotic sensitivity test (AST)

The Kirby-Bauer disc diffusion method, following the standardized protocols and interpretation criteria established by the Clinical and Laboratory Standards Institute (CLSI) was utilized to conduct the AST of selected bacteria [35]. A volume of 50 µl of the bacterial suspension (2.5 × 10⁸ CFU/ml) was inoculated into Mueller-Hinton agar (MHA) flasks, which were then poured into sterile Petri dishes. Once the agar solidified, antibiotic discs from different classes were aseptically added. The plates were then incubated for 24 h. at 37 °C. After the incubation period, the zones of inhibition were measured in millimeters.

### Antibacterial activity assessment using agar well diffusion method

The antibacterial activity of the prepared nanomaterials against the MDR *S.* subsp. *enterica* serovar Typhi ATCC 19214 was assessed using the agar well diffusion method following CLSI recommendations [36]. Bacterial suspensions (2.5 × 10⁸ CFU/ml) were mixed with MHA in plates. A volume of 100 µl of ZnO NPs, CAZ, MTF, CS, and their combination (150 µg/ml in DMSO) was added to 5 mm wells and incubated for 24 h. at 37 °C. Azithromycin was used as the standard antibacterial agent. Inhibition zones were measured at the end of the incubation period.

### Minimum inhibitory concentration (MIC) test

The MIC was estimated using the broth microdilution method [37]. To assess the MICs of the prepared nanomaterials, serial dilutions (1–200 µg/ml) were added to Mueller-Hinton broth (MHB) with a bacterial inoculum (2.5 × 10⁸ CFU/ml) and incubated for 24 h. at 37 °C with agitation at 150 rpm. The inhibition of growth of the treated bacterium was determined spectrophotometrically at 600 nm and compared to the untreated bacterium as well as the azithromycin-treated bacterium, which served as negative and positive controls, respectively.

### Minimum bactericidal concentration (MBC) test

Inoculating MHA plates with 10 µl of inoculant from MICs flasks that were prepared according to the pour plate method, was done to determine the MBCs for the prepared nanomaterials. The total count of bacterial colonies was measured in colony-forming units per milliliter (CFU/ml) following a 24-h incubation period at 37 °C.

### Molecular docking study

The crystal structures of *S. typhi* OmpC (3UU2) and *S. enterica* serovar OMPs (4W4M) were retrieved from the Protein Data Bank (RCSB PDB). The proteins underwent preparation steps including the removal of water molecules, addition of polar hydrogens, and energy minimization. Active binding sites were generated using a site finder in the Molecular Operating Environment (MOE 2019) software (Chemical Computing Group, Montreal, Canada), which served as dummy sites for the binding pocket.

The 3D structures of ZnO NPs, CS, and CAZ were optimized using Gaussian 09 at the B3LYP/6-31G* level. ZnO NPs were modeled as clusters (Zn₄O₄) to simulate nanoscale behavior. Molecular docking was performed using the Triangle Matcher placement technique, with the rigid receptor atoms docked for 100 ns. The GBVI/WSA dG procedures were used for rescoring, with the London dG serving as the scoring function. Multiple poses were generated for each ligand-protein pair, and the top pose was selected for further analysis. 2D and 3D interaction diagrams were created to illustrate how the ligands bind to the active sites of the proteins. These diagrams specifically highlighted the interactions. The docked complexes were examined to identify the interactions between the ligands under investigation and the active site residues of the proteins.

### Cytotoxicity assessment

The anticancer potential of the prepared nanocomposite was evaluated through an MTT assay, comparing it to doxorubicin and sorafenib as reference drugs. Cells were cultured in RPMI-1640 medium with 10% FBS, 100 U/ml penicillin, and 100 µg/ml streptomycin at 37 °C with 5% CO₂, then seeded in 96-well plates (1 × 10⁴ cells/well) for 48 h. After treatment with different compound concentrations for 24 h, 20 µL of MTT (5 mg/mL) was added and incubated for 4 h. Formazan crystals were dissolved using 100 µl DMSO, and absorbance was measured at 570 nm [38, 39].

Cell viability (%) was calculated as:

$$\:=\frac{A\mathrm{570}\:\left(treated\:sample\right)}{A\mathrm{570}\:\left(control\right)}*100$$

## Results and discussion

### Characterization of the nano-formulations

**Fig. 1:**
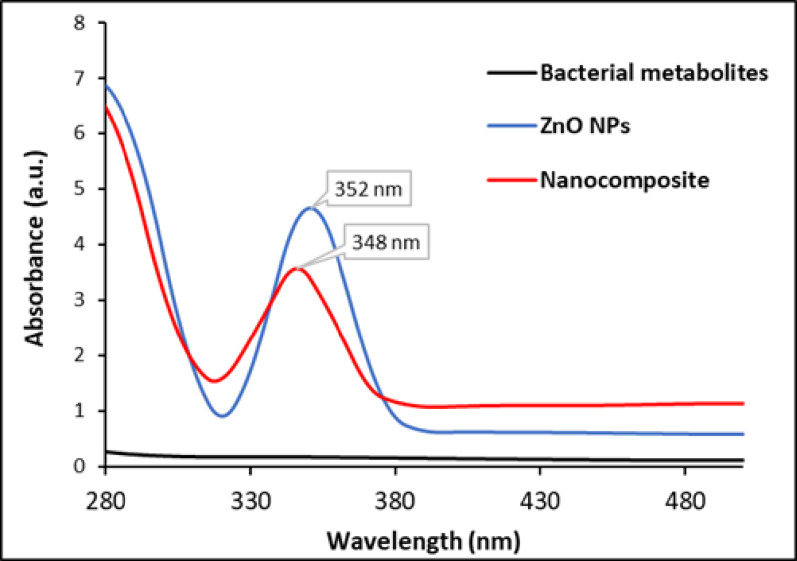
UV-spectra of the cell-free bacterial supernatant, ZnO NPs, and the ZnO/CS/CAZ/MTF composite

Various analytical techniques were utilized to confirm and characterize the formation, structural properties, molecular interactions, morphology, and stability of a synthesized ZnO NPs-based nanocomposite with CAZ, MTF, and CS. The formation of the ZnO NPs and their integration into the nanocomposite were initially verified using UV-Visible spectroscopy. The characteristic absorption peak of ZnO NPs is typically seen at 352 nm, and for ZnO/CS/CAZ/MTF at 348 nm, serving as a reference point (Fig. 1) [40]. The blue shift in the absorption peak position of ZnO upon incorporation of CAZ, MTF, and CS indicates successful interaction and integration of these components within the nanocomposite [41]. Zinc oxide (ZnO) is a wide-bandgap semiconductor with an energy of approximately ≈ 3.37 eV. When reduced to the nanoscale, the confinement of electrons and holes within a small volume results in quantum size effects, which increase the effective bandgap energy of the NPs. Because energy and wavelength are correlated (E = λhc), a decrease in wavelength corresponds to an increase in energy [42]. As a result, when the size of ZnO particles decreases, their absorption peak shifts to a shorter wavelength (a blue shift), which is observed when the NPs are embedded in a polymer [43]. These spectral changes reflect modifications in the electronic environment of ZnO NPs, suggesting possible molecular interactions and complex formation among the constituents [44].

**Fig. 2:**
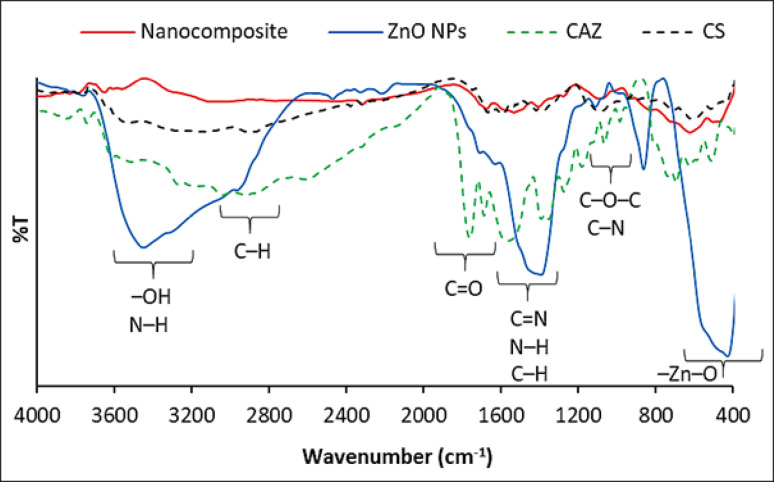
FTIR spectra of ZnO NPs, CS, CAZ, and ZnO/CS/CAZ/MTF nanocomposite

FTIR spectroscopy was used to study the structural characteristics and molecular interactions in the ZnO-based nanocomposite, as shown in Fig. 2. The FTIR spectrum showed several absorption bands confirming the successful inclusion of ZnO, CS, CAZ, and MTF in the nanocomposite matrix. The absorption bands observed in the range of 400–800 cm⁻¹, with a distinct peak at approximately 510 cm⁻¹, are attributed to Zn–O stretching vibrations, confirming the presence and structural retention of ZnO NPs within the composite [45]. A broad absorption band in the 3200–3300 cm⁻¹ region corresponds to overlapping O–H and N–H stretching vibrations originating from hydroxyl and amino groups of CS and MTF, as well as surface hydroxyl groups of ZnO, indicating extensive hydrogen bonding and electrostatic interactions within the nanocomposite matrix [44, 46]. The peak at 2878 cm⁻¹ is assigned to C–H stretching vibrations of aliphatic chains. A strong absorption band detected at 1740–1749 cm⁻¹ is characteristic of carbonyl (C = O) stretching, confirming the presence of CAZ and its β-lactam structure [47, 48]. Additional bands appearing in the 1645–1590 cm⁻¹ region are attributed to C = N stretching and N–H bending vibrations associated with MTF and CS, while the band at approximately 1425 cm⁻¹ corresponds to C–H deformation modes. Peaks observed in the 1028–1092 cm⁻¹ range are assigned to C–O–C and C–N stretching vibrations of the polysaccharide backbone of CS and functional groups of CAZ, further supporting the successful integration of the organic components into the ZnO-based matrix. Notably, slight shifts and changes in the intensity of characteristic –OH, –NH, and C = O bands compared to those of the individual constituents indicate strong intermolecular interactions between ZnO nanoparticles and the organic components, particularly chitosan, consistent with recent literature reports [49]. Comparison with recent studies further confirms the validity of these assignments: previous reports have identified Zn–O stretching in the 400–600 cm⁻¹ region, broad O–H/N–H stretching around 3200–3500 cm⁻¹ for chitosan-based ZnO nanocomposites, and carbonyl C = O bands around 1740–1780 cm⁻¹ for β-lactam antibiotics incorporated in polymer matrices. Peaks in the 1600–1650 cm⁻¹ and 1020–1150 cm⁻¹ ranges, corresponding to C = N, N–H bending, C–N, and C–O–C stretching vibrations, were also consistent with previous reports of chitosan and drug-loaded ZnO systems [50–53]. Overall, the FTIR spectrum, along with UV–Vis spectral evidence, strongly supports the successful fabrication, molecular integration, and structural stability of the ZnO–CS–CAZ–MTF nanocomposite without the formation of new covalent bonds.

**Fig. 3:**
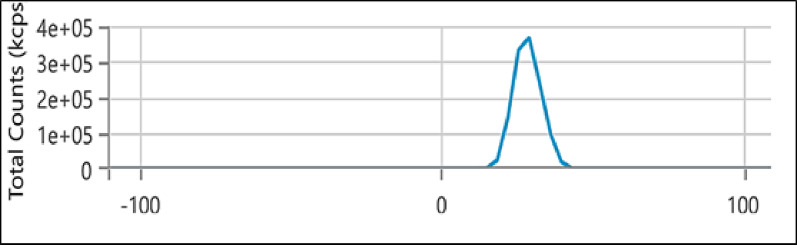
Zeta potential distribution of ZnO/CS/CAZ/MTF nanocomposite

As shown in Fig. 3, the zeta potential measurement of the synthesized ZnO-based nanocomposite revealed a positive surface charge of +28.61 mV. This indicates excellent colloidal stability and strong electrostatic repulsion between particles. Typically ZnO NPs have a negative surface charge due to surface hydroxyl groups, the observed shift to positive values is primarily attributed to the presence of CS, a cationic biopolymer [54, 55]. In addition, CAZ and MTF, both containing ionizable polar groups, may have contributed to the modulation of surface charge by interacting with the nanoparticle surface or the polymer matrix. This enhanced surface potential improves the stability of the nanocomposite and facilitates favorable electrostatic interactions with negatively charged bacterial membranes, potentially enhancing its antibacterial efficacy [56].

**Fig. 4:**
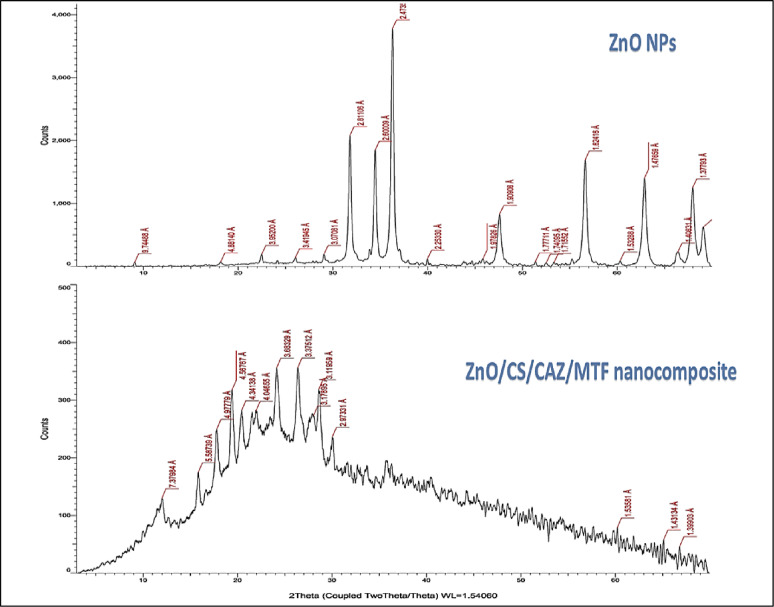
XRD patterns of biosynthesized ZnO NPs and ZnO/CS/CAZ/MTF nanocomposite

The XRD pattern of the biosynthesized pure ZnO NPs showed sharp and well-defined diffraction peaks at 2*θ* values of 31.8°, 34.4°, 36.2°, 47.5°, 56.6°, and 62.8°. These peaks corresponded to the (100), (002), (101), (102), (110), and (103) planes of the hexagonal wurtzite phase, in line with the JCPDS card no. 36–1451 (Fig. 4). The peaks confirmed the successful synthesis of highly crystalline and phase-pure ZnO NPs, with an estimated average crystallite size of approximately ~29.3 nm calculated using the Scherrer equation.

The XRD results of the ZnO/CS/CAZ/MTF nanocomposite showed several distinct peaks at angles of 31.0°, 34.1°, 36.3°, 47.1°, 56.5°, and 62.4°, which correspond to the (100), (002), (101), (102), (110), and (103) crystallographic planes. This confirms the successful integration of ZnO NPs within the ZnO/CS/CAZ/MTF matrix. Upon formulation into a multicomponent composite with CS, CAZ, and MTF, the XRD pattern showed that major ZnO diffraction peaks were retained. This indicates that the core crystal structure of ZnO remained intact after composite formation. However, there were subtle reductions in peak intensity and broadening of specific peaks, particularly at 36.2°, 47.5°, and 56.6°, suggesting a decrease in crystallite size to approximately ~21.6 nm and partial encapsulation of the ZnO NPs within the polymeric matrix. The absence of new diffraction peaks corresponding to crystalline CAZ or MTF suggests that these molecules were incorporated in an amorphous or molecularly dispersed state within the nanocomposite. This is consistent with previous findings that hydrophilic or flexible polymers, such as CS, can effectively embed small-molecule drugs, suppressing their crystallinity while preserving the NPs’ original lattice configuration [57–59].

These structural changes reflect the formation of a core–shell nanostructure or intercalated hybrid system, in which the CS and drug molecules surround or interpenetrate the ZnO. Similar observations have been associated with to the introduction of lattice strain, surface defects, or reduced grain boundaries, all of which can enhance the reactivity, ion release, and antibacterial potency of ZnO-based systems [60–62]. Taken together, the XRD findings demonstrate the successful structural integration of ZnO NPs with CS, CAZ, and MTF, resulting in a stable nanocomposite with refined nanoscale dimensions and enhanced interfacial properties. To the best of our knowledge, this is the first report of a ZnO–CS composite system simultaneously incorporating a β-lactam antibiotic (CAZ) and MTF, marking a novel approach in the development of multifunctional antimicrobial nanomaterials.

**Fig. 5:**
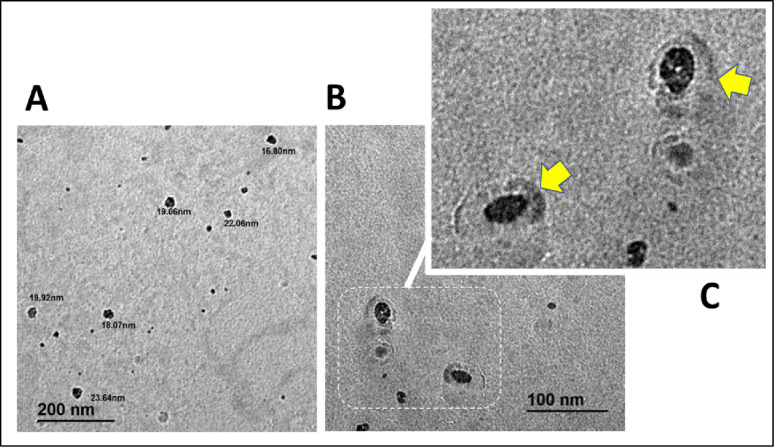
TEM images of ZnO NPs; **A** with scale bar = 200 nm, and ZnO/CS/CAZ/MTF nanocomposite; **B** with scale bar = 100 nm. **C** Magnified part of TEM micrograph of ZnO NPs surrounded by a coat of a mixture of CS, CAZ, and MTF (yellow arrows)

The TEM analysis revealed that the synthesized ZnO/CS/CAZ/MTF nanocomposite consisted of well-dispersed, quasi-spherical NPs with smooth edges (Fig. 5). The observed particle diameters ranged from approximately 9.93 nm to 17.44 nm, indicating nanoscale dimensions ideal for biomedical and antimicrobial applications. These findings confirm the nanostructured nature of the composite and suggest that the CS matrix played a crucial role in preventing agglomeration and stabilizing the particles during synthesis. The consistent uniform morphology and size range observed align with previous studies on biosynthesized ZnO NPs, which typically display spherical or hexagonal shapes and sizes ranging from 20 to 32 nm [19, 63, 64]. The slightly smaller average particle size found in this study may be attributed to the presence of CS as a stabilizing agent and the inclusion of low-molecular-weight drugs such as CAZ and MTF. Although CAZ and MTF are not clearly visible as distinct phases in the TEM image, their existence likely resulted in subtle surface modifications and enhanced particle dispersion. This is corroborated by previous research indicating that drug molecules, when incorporated into ZnO/CS systems, tend to integrate in an amorphous or intercalated state within the biopolymeric network [65, 66].

Moreover, the nanoscale particle size observed by TEM closely matches the crystallite size estimated from XRD analysis (approximately ~21.6 nm), supporting the structural integrity and uniformity of the nanocomposite. These features are particularly beneficial for enhancing surface reactivity, cellular interaction, and antibacterial performance due to the high surface-area-to-volume ratio [67]. This is further supported by the enhanced generation of ROS, efficient Zn²⁺ ion release, and improved interactions with bacterial cell membranes, all of which contribute to the potent antibacterial properties observed in smaller ZnO NPs [68]. Overall, the TEM results confirmed that the ZnO-based nanocomposite maintained a favorable morphology and dispersion after drug incorporation, reflecting the successful formulation of a stable, nanosized antimicrobial delivery system.

### AST pattern

**Fig. 6:**
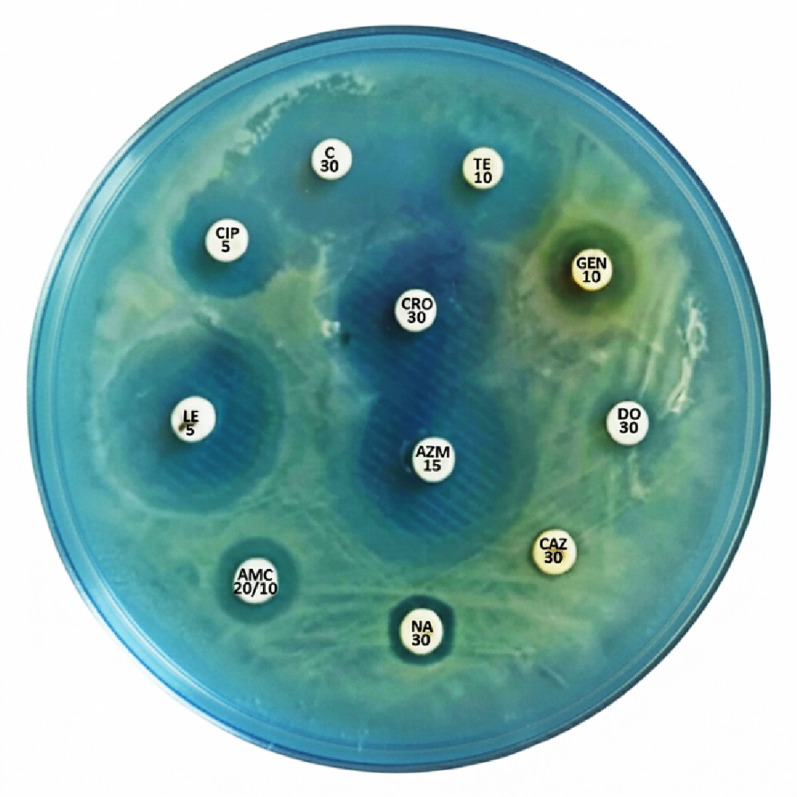
Antibiotic sensitivity test of the ATCC 19214 strain using the disc diffusion technique against various antibiotic discs from different classes. Amoxicillin/clavulanate; (AMC), azithromycin; (AZM); ceftazidime; (CAZ), ceftriaxone; (CRO), chloramphenicol; (C), ciprofloxacin; (CIP), doxycycline; (DO), gentamicin; (GEN), levofloxacin; (LE), nalidixic acid; (NA), and tetracycline; (TE)

The resistance of the strain to several of the tested antibiotics was investigated using the Kirby-Bauer disc diffusion method, following the standardized protocols and interpretation criteria established by the CLSI. This methodology was specifically chosen for standard antibiotics to ensure the accurate classification of the strain as a MDR bacterium based on validated clinical breakpoints. *S.* subsp. *enterica* serovar Typhi ATCC 19214 demonstrates 100% resistance to all tested antibiotic, except for ceftriaxone, levofloxacin, and azithromycin (Table 1; Fig. 6). The strain’s ampicillin resistance is one of its distinguishing features, making it a well-known example of a MDR *Salmonella* strain. Typically, encoded on a plasmid, the ampicillin resistance is accompanied by resistance to streptomycin, chloramphenicol, and sulfanilamide [69]. In the history of *Salmonella* resistance, the strain’s ampicillin resistance is a historical footnote. Although ampicillin was one of the first-line antibiotics used to treat typhoid fever, treatment regimens changed in the 1970 s and 1980 s when MDR strains of the disease appeared. These strains were resistant to ampicillin, trimethoprim-sulfamethoxazole, and chloramphenicol [70]. This is a practical illustration of how the resistance profile of a bacterial strain can directly impact clinical practice and public health. The ampicillin-resistant plasmid is typically not susceptible to the amoxicillin/clavulanate combination due to the presence of extra resistance genes on the plasmid and its overall multidrug resistance. This makes it a valuable control strain for labs examining antibiotic resistance.

The resistance of ATCC 19214 to gentamicin and tetracycline has been documented [71]. A plasmid containing resistance genes for tetracycline, streptomycin, sulfanilamide, and chloramphenicol often plays a role in mediating this resistance. An effective surrogate marker for reduced susceptibility to fluoroquinolones, such as ciprofloxacin and ofloxacin, which were formerly the first-line treatment for typhoid fever, is *S. Typhi* resistance to nalidixic acid [72]. However, azithromycin has proven to be effective against numerous MDR and extensively drug-resistant (XDR) strains of typhoid fever that are resistant to the aforementioned medications; it has emerged as a vital antibiotic for treating the infection. Despite ATCC 19214 being sensitive, new clinical isolates of *S. typhi* especially those from South Asia and other regions are increasingly becoming resistant to azithromycin [73]. With limited treatment options available for typhoid fever, this poses a serious public health hazard.

**Table 1:** Patterns of antibiotic sensitivity

Antibiotic	Concentration (µg/ml)	Diameter of inhibition zones (mm) according to CLSI guidelines			Result (mm)	Response
		*R* ^a^	I	S		
Amoxicillin/clavulanate	20/10	≤ 13	14–17	≥ 18	11	R
Azithromycin	15	≤ 12	–	≥ 13	20	S
Ceftazidime	30	≤ 17	18–20	≥ 21	8	R
Ceftriaxone	30	≤ 12	13–19	≥ 20	21	S
Chloramphenicol	30	≤ 15	16–20	≥ 21	18	I
Ciprofloxacin	5	≤ 15	16–20	≥ 21	15	R
Doxycycline	30	≤ 12	13–15	≥ 16	10	R
Gentamicin	10	≤ 12	13–14	≥ 15	13	I
Levofloxacin	5	≤ 13	14–16	≥ 17	23	S
Nalidixic acid	30	≤ 13	14–18	≥ 19	11	R
Tetracycline	10	≤ 14	15–18	≥ 19	18	I

^a^Resistant (R), Intermediate (I), Susceptible (S)

### Antibacterial activity of the nano-formulations

**Fig. 7:**
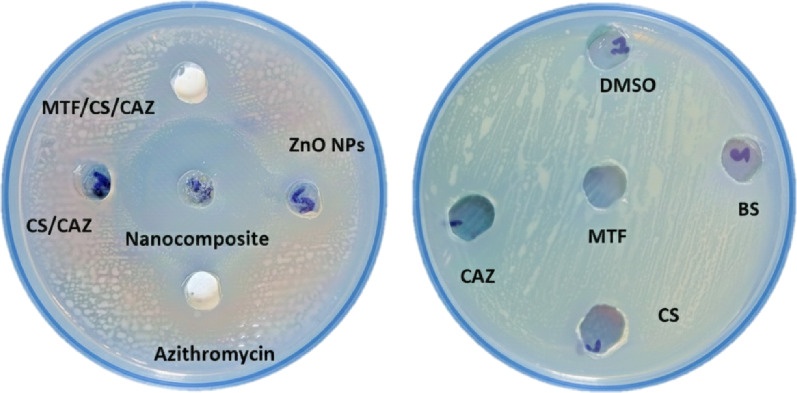
Agar well diffusion test of the prepared materials against the ATCC 19214 strain. Chitosan/ceftazidime (CS/CAZ); metformin/chitosan/ceftazidime (MTF/CS/CAZ); ceftazidime (CAZ); metformin (MTF); chitosan (CS); bacterial metabolites (BS); and the solvent dimethyl sulfoxide (DMSO)

The antimicrobial potential of ZnO NPs, CAZ, MTF, CS, and their combination was evaluated using the agar well diffusion assay at a fixed concentration of 150 µg/ml. This method was chosen instead of disc diffusion to account for the unique physicochemical properties of the prepared nanocomposite materials. The high viscosity of the CS biopolymer and the particulate form of the ZnO NPs could cause paper discs to serve as a barrier, trapping larger molecular complexes within the cellulose matrix and potentially resulting in to underestimated zones of inhibition. The well diffusion technique provides a direct reservoir that facilitates the unimpeded radial diffusion of the viscous nanocomposite and the synergistic release of Zn ions and antibiotics into the agar medium. The robust zones of inhibition observed through the well diffusion method confirm that ZnO/CS/CAZ/MTF effectively diffuses through the aqueous phase of the agar, overcoming the steric hindrances often encountered with traditional disc delivery for macromolecular composites. The results revealed varying antibacterial activity against the MDR strain, with the combined formulation exhibiting the largest inhibition zone, indicating a synergistic interaction. The measured zones of inhibition are displayed in Table 2; Fig. 7. The significantly larger inhibition zone for the combined treatment, ZnO/CS/CAZ/MTF, suggests enhanced antibacterial efficacy, which is consistent with previous studies demonstrating that ZnO NPs increased membrane permeability and facilitated antibiotic uptake, notably for CAZ [74–76]. Moreover, the positive surface charge of CS augments nanoparticle–bacterial interactions, increasing local drug accumulation and promoting ROS generation [77, 78]. Notably, both MTF and CAZ exhibited negligible inhibition zones against the resistant *Salmonella* strain, indicating limited or no observable antibacterial activity when used individually. Although MTF alone showed limited activity, its presence within the combination may contribute to the modulation of bacterial metabolic pathways or act as an adjuvant, enhancing the effects of the other components.

**Table 2:** Inhibition zone diameters (mm) measured for various nanocomposite formulations against the ATCC 19214 strain

Antibacterial agent	Mean of inhibition zone (mm)	Result according to CLSI guidelines	Obstacle response
CS	-ve	R^a^	–
MTF	-ve	R	–
CAZ	12	R	Weak^b^
CS/CAZ	-ve	R	–
MTF/CS/CAZ	17	S	Moderate
ZnO NPs	19	S	Moderate
ZnO/CS/CAZ/MTF	42	S	Very strong
Azithromycin	20	S	Strong

^a^Resistant (R): An inhibition zone of ≤ 12 mm; Susceptible (S): An inhibition zone of ≥ 13 mm

^b^Weak: ≤12 mm; Moderate: 13–19 mm; Strong: 20–39 mm; Very strong: ≥40 mm

**Fig. 8:**
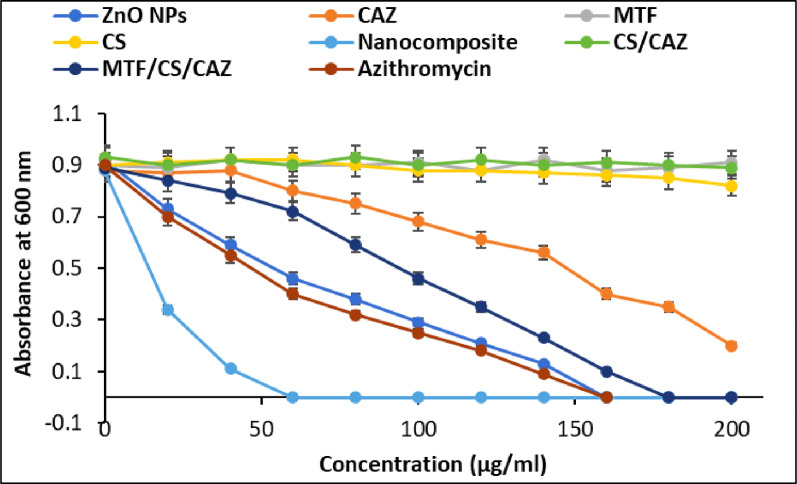
Minimum inhibition concentration of the prepared materials against the ATCC 19214 strain

The MIC of each component and the full combination was determined using the broth microdilution method with a concentration range of 1–200 µg/ml (Fig. 8). As expected, the lowest MIC value was observed for the full nanocomplex, indicating the enhanced potency of the multi-component formulation. The MIC values were as follows: ZnO NPs– 64 µg/ml, CAZ– 32 µg/ml, CS– 80 µg/ml, MTF– >200 µg/ml, and the combination– 8 µg/ml. The MIC values observed for the individual components were as follows: ZnO NPs, 64 µg/ml; CAZ, 32 µg/mL; CS, 80 µg/ml; and MTF,>200 µg/ml. In contrast, the ZnO/CS/CAZ/MTF nanocomposite exhibited identical MIC and MBC values of 8 µg/ml against MDR *Salmonella enterica*, indicating that the concentration required to inhibit bacterial growth is the same as that needed for bacterial killing. In clinical microbiology, an MBC/MIC ratio of ≤ 4 is the definitive hallmark of a bactericidal agent, whereas a ratio > 4 indicates bacteriostatic activity. The observed ratio of 1 suggests that the ZnO/CS/CAZ/MTF nanocomposite exerts a rapid and irreversible lethal effect upon reaching the threshold concentration [79]. This indicates a concentration-dependent bactericidal mechanism, rather than a time-dependent effect, under the in-vitro experimental conditions. Unlike traditional time-dependent β-lactams, where efficacy relies on prolonged exposure, the synergistic action of the NPs and cationic CS likely triggers an immediate loss of membrane integrity and acute oxidative stress, ensuring that inhibitory and bactericidal endpoints are reached simultaneously [80].

Notably, the TEM analysis confirmed the synthesis of fine, quasi-spherical ZnO NPs with diameters ranging from 9.93 nm to 17.44 nm. This small particle size is a critical determinant of the observed antibacterial potency. Particles in this size range (sub-20 nm) are known to exhibit superior antimicrobial activity because they can effectively bypass the physical barriers of the Gram-negative cell envelope [81]. The small dimensions specifically, facilitate the internalization of the NPs or their localization within the periplasmic space. This promotes the localized release of Zn^2+^ ions and the generation of ROS. Furthermore, the high surface-to-volume ratio inherent in this size range enhances the synergistic interaction with CS and CAZ. It provides more active sites for the formation of the quadruple-component complex, ultimately leading to the low MIC value of 8 µg/ml recorded against the MDR *S. Typhi* isolate.

Individually, each component exhibits a distinct mechanism of antimicrobial action: ZnO NPs disrupt bacterial membranes and induce oxidative stress via ROS generation [82]; CAZ acts by inhibiting peptidoglycan synthesis in bacterial cell walls [83]; CS increases bacterial membrane permeability and exhibits its own bacteriostatic effects [84, 85].

MTF, though minimally active on its own, has been reported to facilitate drug delivery and enhance the activity of metal-based complexes [86]. This significant decrease in MIC indicates a synergistic antibacterial effect, where the unique mechanisms of each component work together. ZnO NPs disrupt bacterial membrane integrity and stimulate ROS production; CAZ hinders cell wall synthesis; CS enhances drug retention and boosts permeability. Although MTF is mostly inactive on its own, its presence may enhance molecular interactions or intracellular delivery. The significant decrease in MIC confirms that the composite formulation effectively leverages the complementary actions of each component to address antimicrobial resistance and achieve higher efficacy at lower doses. These results are consistent with prior research showing that ZnO-based nanocomposites, which include antibiotics and polymers, provide enhanced antimicrobial performance through multifaceted synergy [82, 85, 86]. The MIC data confirm the synergistic nature of the combined formulation, which required a significantly lower concentration to inhibit bacterial growth. This observation is consistent with previous research demonstrating that CS-capped ZnO NPs have superior bactericidal activity, and that ZnO NPs enhance antibiotic penetration and ROS production [77, 78, 87]. Spectrophotometric measurements at 600 nm further supported these findings, showing a significant reduction in optical density for the combination treatment compared to controls and single-agent treatments. The growth inhibition of the combination was also comparable to or better than that of penicillin (positive control), indicating its potential as an alternative or adjunctive therapy.

The superior antibacterial performance of the complex may be attributed to its multi-targeted mode of action. This includes membrane disruption by ZnO NPs and CS [88], release of zinc ion (Zn²⁺) which disturb membrane integrity and protein function [75], overproduction of ROS, leading to oxidative damage [78], enhanced antibiotic uptake, particularly of CAZ [75, 76], and metabolic disturbance, possibly influenced by MTF. This synergistic interaction allows the nanocomplex to overcome specific forms of antimicrobial resistance, particularly in *Salmonella*, where conventional antibiotics alone are often ineffective. CS enhances the antibacterial effect of CAZ against *Salmonella* by increasing bacterial membrane permeability and facilitating greater antibiotic uptake [89–91]. Additionally, it improves drug stability and enables sustained release when formulated as NPs or microparticles [90, 92, 93]. These synergistic effects lead to lower MICs and larger inhibition zones compared to CAZ alone [91, 93], which supports the observed enhanced efficacy in the current study.

The MIC value of 8 µg/ml for the ZnO/CS/CAZ/MTF nanocomposite is relatively low compared to the typical clinical serum concentrations and the established clinical benchmarks of CAZ achieved following standard intravenous dosing, which can reach to 60–170 µg/ml [94]. Furthermore, because CAZ constitutes only a fraction of the total nanocomposite mass, the effective concentration of the antibiotic required for bacterial inhibition is drastically reduced compared to monotherapy. This synergistic potency suggests that the nanocomposite could achieve clinical efficacy at lower antibiotic dosages, thereby minimizing systemic toxicity and the risk of further resistance development. The incorporation of MTF into the ZnO-NPs-based complex appears to enhance antibacterial activity against multidrug-resistant *Salmonella* through several complementary mechanisms. MTF, a biguanide compound, has been reported to form coordination complexes with metal ions, improving their delivery and bioavailability [95, 96]. When combined with ZnO-NPs, MTF may promote greater bacterial membrane penetration, contribute to enhanced ROS-mediated damage, and improve biofilm inhibition [95, 97]. These synergistic interactions likely contribute to the observed reduction in MIC values and enhanced inhibition zones in our study, in agreement with previous reports combining ZnO-NPs with other antimicrobial agents [98–100].

### Molecular docking investigations

The superior docking scores of Ceftazidime align with its role as a β-lactam antibiotic targeting penicillin-binding protein (PBPs). Its strong interactions with GLU109 (3UU2) and LYS73 (4W4M) disrupt OMP integrity, facilitating cell lysis [101]. CS’s H-bonding with ASP residues corroborates its known membrane-disruptive properties through electrostatic interactions with bacterial surfaces [25]. ZnO NPs utilize metal coordination with ASP residues to induce oxidative stress and membrane damage [16]. The synergy in the nanocomposite likely stems from multi-target inhibition: ZnO NPs compromise membrane integrity, chitosan enhances drug permeability, and ceftazidime inhibits cell-wall synthesis [102]. The 42 mm inhibition zone for the ZnO/CS/CAZ/MTF nanocomposite confirms synergy. MTF may enhance activity by downregulating bacterial efflux pumps [30], while CS improves ceftazidime’s bioavailability [103]. The low activity of CAZ alone (12 mm) suggests resistance in the tested strain, which is overcome by the nanocomposite-enhanced delivery. This aligns with studies showing that chitosan-ZnO composites restored susceptibility to MDR *Salmonella* [51].

**Fig. 9:**
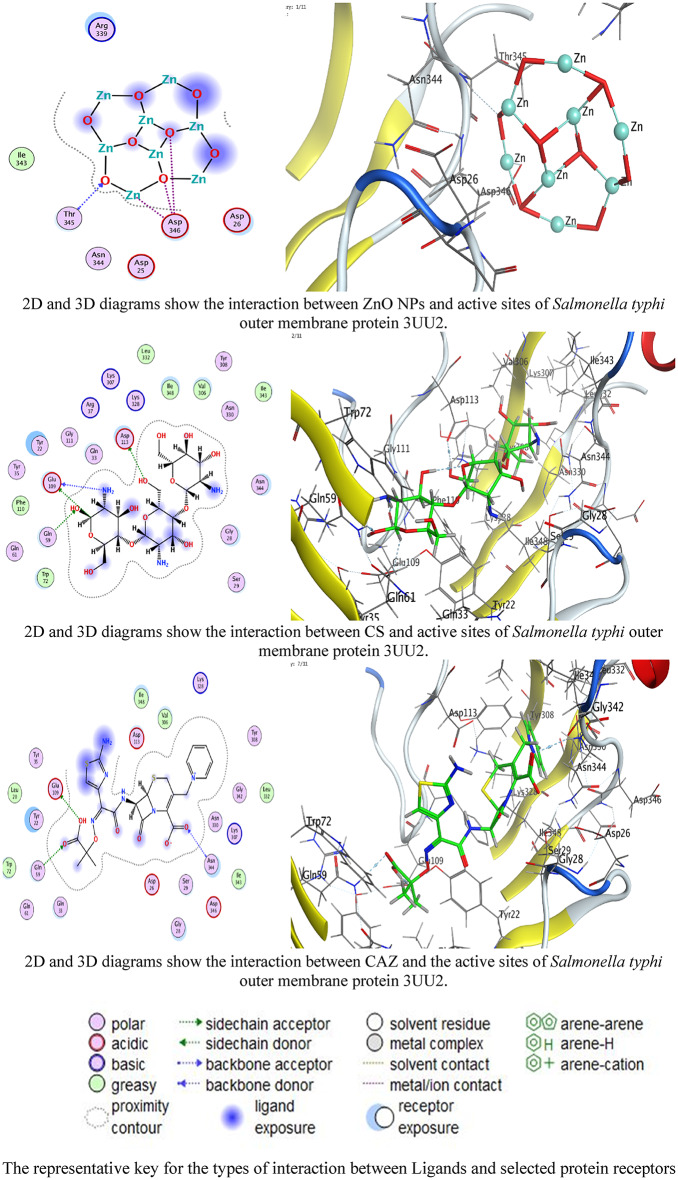
2D and 3D diagrams show the interaction between ZnO NPs, CS, and CAZ and the active sites of *Salmonella typhi* outer membrane protein 3UU2

**Fig. 10:**
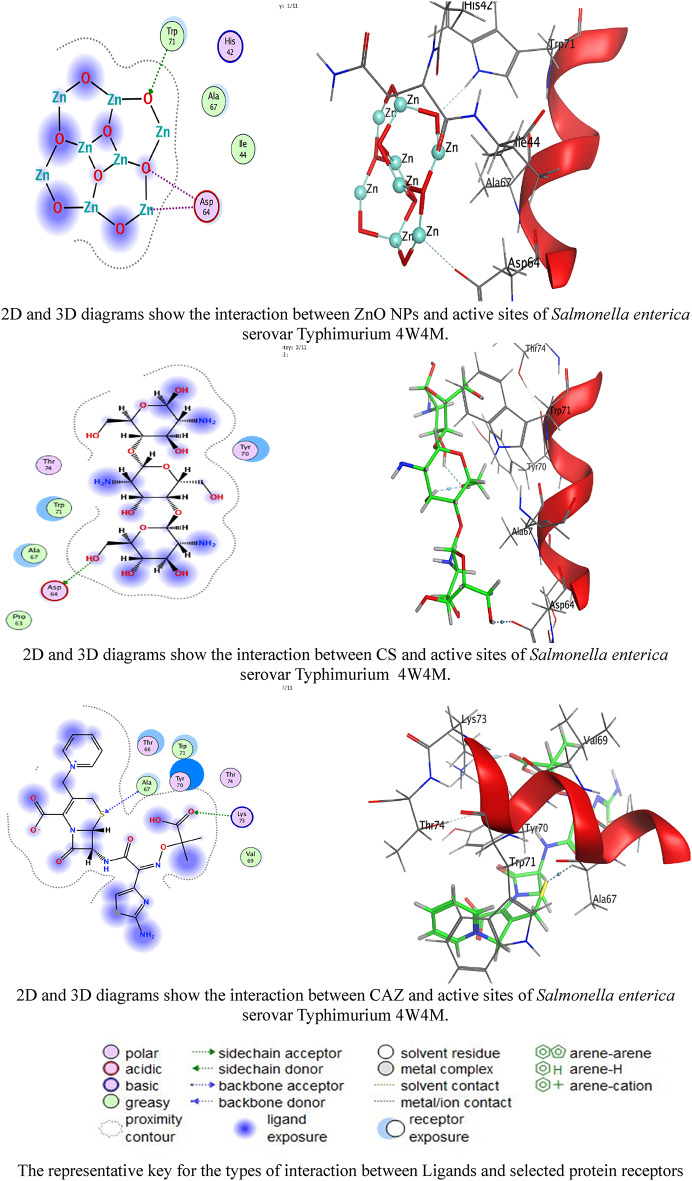
2D and 3D diagrams show the interaction between ZnO NPs, CS, and CAZ and the active sites of *Salmonella enterica* serovar Typhimurium 4W4M

Our docking scores for CAZ against 3UU2 (−8.13574) are consistent with its reported efficacy against Gram-negative bacteria (Tables 3, 4, 5 and 6; Figs. 9 and 10) [4]. The ionic interactions of ZnO NPs with ASP residues align with studies showing that Zn²⁺ disrupts OMPs via competitive binding [104]. The synergy of the nanocomposite parallels findings where CS-coated NPs enhanced antibiotic penetration [105].

Ligands may bind strongly in silico but lack bioavailability or functional activity. For example:

- Chitosan’s large size and hydrophilicity limit its diffusion through agar in well-diffusion assays.
- CAZ may bind OMPs but fail to reach its primary target (penicillin-binding proteins, PBPs) due to permeability barriers.

The discrepancy between positive molecular docking results (favorable binding energies and interactions) for CS and CAZ and their zero or low antibacterial activity in laboratory assays arises from fundamental differences between computational predictions and real-world biological complexity.

*Docking focus*: Docking predicts binding affinity to a single isolated target (e.g., outer membrane proteins OmpC/OmpF in *Salmonella*). It does not account for.

- Cell envelope complexity: Gram-negative bacteria like *Salmonella* have multiple barriers (outer membrane, peptidoglycan, inner membrane). Ligands must penetrate all layers to reach intracellular targets.
- Efflux pumps: Bacteria expel antibiotics via efflux systems (e.g., AcrAB-TolC in *Salmonella*), reducing intracellular drug concentration. Docking ignores this.
- Enzymatic degradation: CAZ is vulnerable to β-lactamases (e.g., extended-spectrum β-lactamases, ESBLs), which hydrolyze its β-lactam ring. Docking cannot model enzymatic inactivation.

### Static vs. dynamic systems

- Docking simulates a static protein-ligand interaction in a vacuum.
- In reality:
- Proteins are dynamic, with conformational changes affecting ligand binding.
- Cellular conditions (pH, ion concentrations, crowding) alter binding kinetics.

### Biological barriers to antibacterial activity

#### Chitosan-specific challenges

*Poor solubility/diffusion*: CS is insoluble at neutral pH (used in agar assays), forming aggregates that cannot diffuse through agar to reach bacteria.

*Mechanism mismatch*: Docking predicts binding to OMPs, but chitosan’s primary antibacterial mechanism requires.

- Direct contact with bacterial membranes (disrupting integrity via electrostatic interactions).
- In agar assays, chitosan cannot penetrate the agar matrix to contact bacteria effectively.

#### Ceftazidime-specific challenges

*Resistance mechanisms*: The tested *Salmonella* strain likely expresses.

- β-lactamases: Enzymes that hydrolyze CAZ before it reaches PBPs.
- Porin mutations: Reduced expression of OmpC/OmpF porins, limiting CAZ uptake.
- Efflux pumps: Active extrusion of CAZ from cells.

#### Role of the nanocomposite in overcoming barriers

The ZnO/CS/CAZ/MTF nanocomposite showed potent activity (42 mm inhibition zone) because:

- ZnO NPs: Disrupt membrane integrity via ROS generation and metal coordination, enhancing permeability.
- Chitosan: Acts as a carrier, improving CAZ’s solubility and adhesion to bacterial surfaces.
- Metformin: Inhibits efflux pumps and β-lactamase expression, potentiating ceftazidime.
- Synergy: The nanocomposite bypasses individual limitations (e.g., chitosan’s poor diffusion, ceftazidime’s degradation).

**Table 3:** Docking scores and energies of ZnO NPs, CS, and CAZ against *Salmonella Typhi* osmoporin(OmpC): an outer membrane protein (PDB ID: 3UU2)

Agent	Docking Score (S)	rmsd_refine	E_conf	E_place	E_score1	E_refine	E_score2
ZnO NPs	−3.53516	1.6591531	−1122.02	−28.7278	−6.20773	−13.7651	−3.53516
CS	−7.17274	2.4780273	258.4574	−86.4263	−12.0304	−42.0456	−7.17274
CAZ	−8.13574	1.7821292	34.24772	−83.7483	−10.5897	−46.759	−8.13574

**Table 4:** Docking scores and energies of ZnO NPs, CS, and CAZ against *Salmonella Enterica* subsp. *Enterica* serovar typhimurium (PDB ID: 4W4M)

Agent	Docking Score (S)	rmsd_refine	E_conf	E_place	E_score1	E_refine	E_score2
ZnO NPs	−4.19742	2.6274469	−1122.07	−26.6811	−2.51933	−24.3852	−4.19742
CS	−5.12714	2.7264142	252.09	−38.6774	−7.9302	−23.2306	−5.12714
CAZ	−5.54416	2.1023273	35.73411	−48.5309	−7.74437	−28.143	−5.54416

**Table 5:** Interaction of ZnO NPs, CS, and CAZ with the structure of *Salmonella Typhi* outer membrane protein (PDB ID: 3UU2)

Agent	Atom involved	Residue	Interaction type	Distance (Å)	Binding Energy (kcal/mol)
ZnO NPs	O 11	N THR 345 (A)	H-acceptor	3.07	−0.7
	Zn 10	OD2 ASP 346 (A)	Metal	2.58	−1.9
	O 3	OD2 ASP 346 (A)	Ionic	4.01	−0.5
	O 5	OD2 ASP 346 (A)	Ionic	3.17	−3.5
CS	O 16	OD1 ASP 113 (A)	H-donor	3.17	−1.0
	N 28	O GLU 109 (A)	H-donor	3.02	−0.7
	C 59	OE2 GLU 109 (A)	H-donor	3.39	−0.5
	O 14	NE2 GLN 59 (A)	H-acceptor	2.96	−2.0
CAZ	O 8	OE2 GLU 109 (A)	H-donor	2.94	−5.1
	O 5	N ASN 344 (A)	H-acceptor	3.52	−0.8
	O 10	NE2 GLN 59 (A)	H-acceptor	2.96	−0.7

**Table 6:** Interaction of ZnO NP, CS, and CAZ with the structure of *Salmonella Enterica* subsp. *Enterica* serovar typhimurium (PDB ID: 4W4M)

Agent	Atom involved	Residue	Interaction type	Distance (Å)	Binding energy (kcal/mol)
ZnO NPs	O 15	NE1 TRP 71 (A)	H-acceptor	2.98	−0.7
	Zn 13	OD2 ASP 64 (A)	Metal	2.58	−2.1
	O 6	OD2 ASP 64 (A)	Ionic	3.36	−2.5
Chitosan	O 20	OD2 ASP 64 (A)	H-donor	3.02	−0.9
Ceftazidime	S 1	CA ALA 67 (A)	H-acceptor	3.64	−0.7
	O 10	NZ LYS 73 (A)	H-acceptor	3.12	−3.6

### In vitro cytotoxicity evaluation of ZnO/CS/CAZ/MTF nanocomposite

**Fig. 11:**
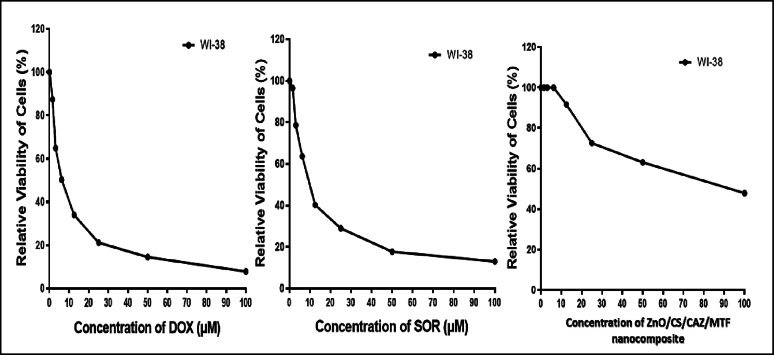
Dose-dependent cytotoxic effect of ZnO/CS/CAZ/MTF nanocomposite on WI-38 lung fibroblast cells determined via MTT assay

The cytotoxic potential of the synthesized ZnO/CS/CAZ/MTF nanocomposite was assessed using the MTT assay against the human lung fibroblast cell line (Table 7; Fig. 11). The assay measures mitochondrial metabolic activity as an indicator of cell viability, with viable cells reducing MTT to insoluble formazan crystals [38]. The composite exhibited an IC₅₀ value of 84.26 ± 4.2 µg/ml, indicating moderate cytotoxicity and acceptable biocompatibility toward normal human cells. In comparison, ZnO NPs alone have been reported to induce dose-dependent cytotoxic effects due to ROS generation and membrane disruption, with IC₅₀ values ranging from 50 to 100 µg/ml depending on particle size and surface modification [106]. CS, being a naturally derived, biocompatible polymer, has minimal cytotoxic effects and is commonly used in biomedical formulations [107]. On the other hand, CAZ, while generally safe for systemic use, may induce oxidative stress at high concentrations when used in nanoparticulate systems [108]. MTF, primarily known for its antidiabetic activity, exhibits low cytotoxicity and has even shown protective effects on some normal cell lines due to its antioxidant and anti-inflammatory actions [109, 110]. The relatively higher IC₅₀ value of the ZnO/CS/CAZ/MTF nanocomposite suggests that the inclusion of CS and MTF may attenuate the cytotoxicity induced by ZnO NPs, possibly through stabilization of the particle surface and reduction of free Zn²⁺ ion release [111, 112]. Furthermore, the absence of significant morphological changes in treated WI-38 cells at sublethal doses supports the composite’s biocompatibility, making it a promising candidate for safe biomedical applications, particularly antibacterial therapy.

**Table 7:** IC₅₀ values of different compounds on WI-38 cells

Compound	In vitro cytotoxicity IC_50_ (µg)^a^
	WI-38
DOX	6.72 ± 0.5
SOR	10.65 ± 0.8
ZnO/CS/CAZ/MTF nanocomposite	84.26 ± 4.2

*DOX* Doxorubicin, *SOR* Sorafenib

^a^IC_50_(µg/ml): 1–10 (very strong), 11–20 (strong). 21–50 (moderate). 51–100 (weak) and above 100 (non-cytotoxic)

### Limitations of the study

One limitation of this study is that the antibacterial mechanisms were based on in-vitro and in-silico experiments without testing in animals. Cytotoxicity was assessed using only one normal cell line, and only one MDR *Salmonella* strain was evaluated. It is recommended to conduct a broader evaluation against multiple clinical isolates for generalization of the results. Additionally, the long-term stability, distribution, and persistence of the nanocomposite in the body (bioavailability and pharmacokinetics), were not assessed. Future studies should include animal models, multiple cell types, and diverse bacterial isolates to confirm these findings.

## Conclusion

This study highlights the successful development of a ZnO-based nanocomposite loaded with CAZ, CS, and MTF. Quantitative evaluation revealed a potent synergistic bactericidal effect against the MDR *Salmonella enterica* subsp. *enterica* serovar Typhi ATCC 19214 with an enlarged inhibition zone (42 mm) and identical MIC and MBC values (8 µg/ml). The high efficacy of this treatment is due to a multi-targeted mechanism that suppresses traditional resistance barriers; CS and ZnO NPs cause membrane permeabilization through electrostatic and oxidative stress, allowing CAZ to enter the cell and inhibit cell-wall synthesis. Meanwhile, MTF disrupts metabolic homeostasis. Molecular docking supports this synergy, showing that the treatment’s potency is driven by strong binding affinities to *Salmonella* outer membrane proteins (OMPs) through metal coordination (ZnO), H-bonding (CAZ), and electrostatic interactions (CS). While individual components showed strong in-silico binding, their lack of in-vitro efficacy confirms that overcoming MDR requires the simultaneous mechanical and biochemical attack provided by the composite system. By bridging experimental microbiology with molecular modeling, this study underscores that overcoming multidrug resistance requires multi-targeted strategies that disrupt bacterial homeostasis at several levels simultaneously—an aspect that molecular docking describes structurally but cannot fully predict dynamically. These findings support the potential of this multicomponent system as a promising alternative to conventional monotherapies. Future research will focus on evaluating in-vivo biosafety, anti-biofilm activity, and the development of targeted delivery platforms to move this technology toward clinical application.

## Data Availability

The datasets used and/or analyzed during the current study are available from the corresponding author on reasonable request.

## Abbreviations

AST: Antibiotic sensitivity test
E_conf: Energy conformer
E_place: Score of the placement phase
E_refine: Score refinement
ESBLs: Extended-spectrum β-lactamases
FTIR: Fourier transform infrared spectroscopy
IC₅₀: Cytotoxic concentration 50%
MBC: Minimum bactericidal concentration
MDR: Multidrug-resistant
MIC: Minimum inhibition concentration
MOE: Molecular operating environment
OMPs: Outer membrane proteins
TEM: Transmission electron microscopy
UV-Vis: Ultraviolet-visible spectroscopy
XRD: X-ray diffraction
ZnO NPs: Zinc oxide nanoparticles
ZnO/CS/CAZ/MTF: ZnO NPs/ceftazidime/metformin/chitosan nanocomposite
